# The implementation of value-based healthcare: a scoping review

**DOI:** 10.1186/s12913-022-07489-2

**Published:** 2022-03-01

**Authors:** Dorine J. van Staalduinen, Petra van den Bekerom, Sandra Groeneveld, Martha Kidanemariam, Anne M. Stiggelbout, M. Elske van den Akker-van Marle

**Affiliations:** 1 Medical Decision Making, Department of Biomedical Data Sciences, Leiden University Medical Center, PO Box 9600, Albinusdreef 2, 2300RC Leiden, The Netherlands; 2 Institute of Public Administration, Leiden University, Turfmarkt 99, 2511 DP The Hague, The Netherlands

**Keywords:** Value-based healthcare, Implementation, Delivery of health care, Hospitals, Health policy

## Abstract

**Background:**

The aim of this study was to identify and summarize how value-based healthcare (VBHC) is conceptualized in the literature and implemented in hospitals. Furthermore, an overview was created of the effects of both the implementation of VBHC and the implementation strategies used.

**Methods:**

A scoping review was conducted by searching online databases for articles published between January 2006 and February 2021. Empirical as well as non-empirical articles were included.

**Results:**

1729 publications were screened and 62 were used for data extraction. The majority of the articles did not specify a conceptualization of VBHC, but only conceptualized the goals of VBHC or the concept of value. Most hospitals implemented only one or two components of VBHC, mainly the measurement of outcomes and costs or Integrated Practice Units (IPUs). Few studies examined effects. Implementation strategies were described rarely, and were evaluated even less.

**Conclusions:**

VBHC has a high level of interpretative variability and a common conceptualization of VBHC is therefore urgently needed. VBHC was proposed as a shift in healthcare management entailing six reinforcing steps, but hospitals have not implemented VBHC as an integrative strategy. VBHC implementation and effectiveness could benefit from the interdisciplinary collaboration between healthcare and management science.

**Trial registration:**

This scoping review was registered on Open Science Framework https://osf.io/jt4u7/ (OSF | The implementation of Value-Based Healthcare: a Scoping Review).

**Supplementary Information:**

The online version contains supplementary material available at 10.1186/s12913-022-07489-2.

## Introduction

The plea to change from a volume-driven into a value-driven or value-based healthcare (VBHC) originated in the 90s [1–5]. This change implies that healthcare systems focus increasingly on quality of care rather than volume of care. Attention for a change toward a value-driven healthcare system accelerated when Porter & Teisberg introduced value-based healthcare (VBHC): a new strategy for how healthcare should be delivered and measured [6]. VBHC focuses on delivering value for patients and value is defined as health outcomes achieved per dollar spent. Value can increase by lowering healthcare costs or improving outcomes, or both.

Since its introduction by Porter & Teisberg in 2006 [6], VBHC has received growing attention, and healthcare organizations in several countries are changing their strategies towards VBHC. VBHC was operationalized by Porter & Teisberg into six components that were assumed to be mutually reinforcing: organize care into Integrated Practice Units (IPUs), measure outcomes and costs for every patient, move to bundled payments for care cycles, integrate care delivery across separate facilities, expand excellent services across geography, and build an enabling information technology platform. Porter & Teisberg presented minimal guidance, though, on which strategies should be deployed for the implementation of VBHC and under which circumstances strategies were most suitable.

Ambiguity exists regarding both the conceptualization and the implementation of VBHC [7–9] which makes it difficult to share best practices or compare across healthcare organizations. VBHC conceptualization refers to how authors define VBHC, while VBHC implementation refers to what activities are executed in hospitals under the umbrella of VBHC.

Implementation strategies refer to *how* VBHC implementation is put into practice and include *“approaches or techniques used to enhance the adoption, implementation, sustainment, and scale-up (or spread) of an innovation”* [10]. An overview of conceptualization, implementation and implementation strategies used in the context of VBHC is needed and missing in the current literature.

We therefore aim to provide an overview regarding the conceptualization and implementation of VBHC as introduced by Porter & Teisberg, and of the implementation strategies used. Furthermore, we describe the effects of the implemented VBHC components and the used implementation strategies. To this end we addressed the following research questions:

1. How is VBHC conceptualized in the current VBHC literature?
2. What components of VBHC are implemented or proposed to be implemented, and what effects of implementing these components are reported?
3. What strategies are used or proposed to implement VBHC and what effects of these strategies are described?

## Methods

### Study design

We conducted a scoping review in accordance with the methodology of the Joanna Briggs Institute and the framework of Arksey and O’Malley [11]. The Preferred Reporting Items for Systematic Reviews and Meta-Analyses extension for Scoping Reviews (PRISMA-ScR) were followed [12]. This scoping review was registered on Open Science Framework. Since research on VBHC is heterogeneous and methodologies to study VBHC differ, a scoping review was suitable to answer the broad research questions in this study.

### Search strategy

We searched multiple electronic databases: EMBASE, Pubmed and Web of Science. All databases were searched for the same time frame, starting January 2006 – the year in which Porter and Teisberg coined VBHC – up to February 2021.

A Medical Subject Heading (MeSH) term in referring to VBHC Pubmed does not exist, but the MeSH term Value-Based Health Insurance does and was therefore added to the search. Due to the lack of a VBHC MeSH term, multiple search terms were used. The search terminology was set up as follows: (“Value-Based Health Insurance”[Mesh] OR “value based care”[tw] OR “value based healthcare”[tw] OR “value based health care”[tw] OR “valuebased care”[tw] OR “valuebased healthcare”[tw] OR “valuebased health care”[tw] OR “value-based care”[tw] OR “value-based healthcare”[tw] OR “value-based health care”[tw] OR “VBHC”[tw]).

### Study eligibility

The main subject of the included articles needed to be VBHC. Full text articles in English that described the implementation of VBHC in a hospital setting or healthcare system were included. In order to create a complete comprehensive overview of VBHC components that have been implemented and of implementation strategies used in VBHC literature, we included empirical as well as non-empirical articles. Literature reviews were excluded, but their references were evaluated for eligible articles.

As described above, the terms ‘value-driven care’ and ‘value-based care’ were introduced before Porter and Teisberg introduced ‘value-based healthcare’ in 2006. To stay close to the ideas of Porter and Teisberg [6], the selection of articles was narrowed down to studies that explicitly used the term VBHC or the term Value-Based Care with an explicit reference to Porter & Teisberg. Articles on related concepts such as bundled payments, or broader conceptualizations such as population health, that did not use these terms were not included.

### Study selection

The articles from the search were exported to EndNote after which duplicates were removed. Eligibility screening was done using the online program Rayyan [13]. First, titles and abstracts were screened by two reviewers (D.S. and M.K.) independently, who discussed disagreements after every 200 screened articles. If agreement was not reached, the titles in question were discussed with a third, or when needed, a fourth reviewer (A.S. and E.A.). Full text screening was done independently by two researchers (D.S. and A.A.).

### Data extraction and synthesis

Data extraction and evaluation were performed by three reviewers (D.S., E.A., P.B.). Screening of a sample of the data extraction was performed independently by a fourth author (A.S.). We used the following extraction fields to organize and summarize study findings: author, year, country, VBHC conceptualization, VBHC implementation, VBHC component, implementation strategies, evaluation focus, reported effects and study design. The operationalization of the different data extraction fields is presented in Supplementary Table S1. Subsequently, these data were regrouped to answer the research questions. Data in the field VBHC conceptualization was categorized to indicate how VBHC is conceptualized in the current literature (research question 1). Data from the field VBHC implementation and VBHC component were used to identify what is implemented or proposed to be implemented as VBHC (research question 2). Lastly, the remaining three fields were used to indicate implementation strategies and their effects (research question 3).

## Results

**Fig. 1:**
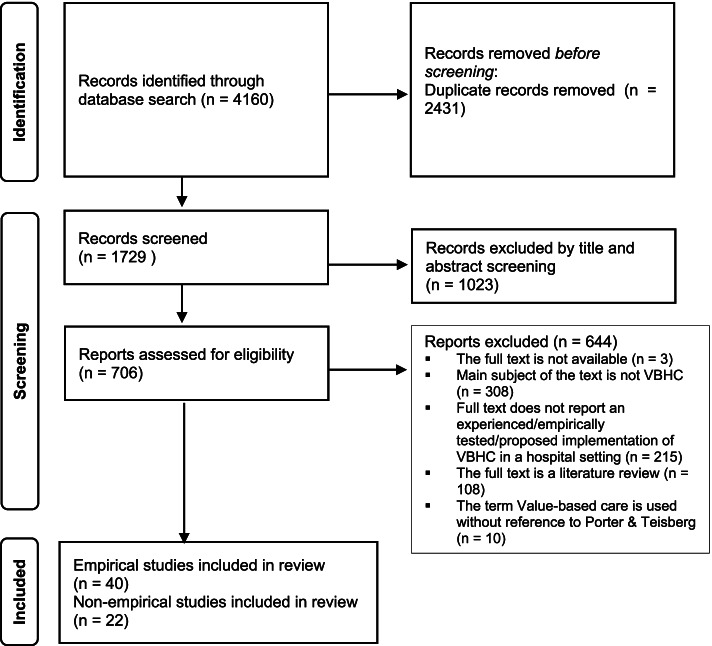
Flowchart of search results and record selection

The initial database search identified 4160 references. After deduplication, 1729 references were eligible for title/abstract screening. The title/abstract screening resulted in 706 full text articles. After screening these, we selected 62 original articles for inclusion: 40 empirical and 22 non-empirical, originating from the United States (*n* = 30), the Netherlands (*n* = 9), the United Kingdom (*n* = 7) and other countries (*n* = 16). An overview of the article selection is shown in Fig. 1.

### VBHC conceptualization

Supplementary Tables S2 and S3 present an overview of the included studies. In fourteen of the 40 empirical articles that described an implementation of VBHC, VBHC was fully conceptualized [8, 9, 14–25](i.e. including a theoretical approach in combination with a conceptualization of the value or goals). In 15 articles, the concept of VBHC was defined by reference to its value or its goals only, without defining the VBHC concept. Six conceptualized value in VBHC [26–31], six conceptualized goals of VBHC [7, 32–36] and three conceptualized both value and goals in VBHC [37–39]. The remaining articles (*N* = 11) did not include a conceptualization of VBHC [40–50].

As to the 22 non-empirical articles, only two fully conceptualized the concept of VBHC [51, 52]. In 6 articles, the concept of VBHC was defined solely by reference to its value or its goals: three articles conceptualized only the value in VBHC [53–55] and three articles only the goals of VBHC [56–58]. The remaining fourteen articles did not conceptualize either the concept of VBHC, value, or goals [59–72].

### Implemented VBHC components and effects

Supplementary Table S2 shows that in the empirical studies the most frequently implemented VBHC component was ‘measure outcomes and costs for every patient’ (*N* = 31)[9, 14–18, 20–28, 30–32, 35–40, 42, 44–49]. In general, patient-reported outcome measures (PROMs) were used: provider-reported experience measures were included only once in the outcome measurement set [36]. Time Driven Activity Based Costing (TDABC) was described in six studies [27, 28, 30, 31, 40, 46]. The second most implemented component was ‘organize care into IPUs’ (*N* = 12)[14, 18–21, 26, 33, 35, 36, 40, 44, 47]. Often, these studies described the implementation of care pathways. Five articles described the component ‘building an enabling information technology platform’, e.g. an interactive application to collect patient experiences or a dashboard [16, 17, 22, 29, 36]. Two studies described the implementation of ‘move to bundled payments for care cycles [34, 50]’. Lastly, four articles described the ‘integration of care delivery across separate facilities’ [7, 25, 42, 43], often in the form of an Accountable Care Organization, a collaboration between regional healthcare services or a roadmap to reform healthcare delivery.

In the non-empirical literature, the most frequently mentioned component was ‘measuring costs and outcomes for every patient’ (*N* = 16)[51–57, 59, 60, 62, 65, 67, 69–72]. The second most often mentioned was ‘moving to bundled payments for the full cycle of care’ (*N* = 7)[51, 55, 59, 61, 64, 68, 69]. Six articles described the component ‘organizing care into IPUs’ [52, 55, 56, 58, 66, 69]. Other articles (*N* = 5) mentioned the ‘integration of care delivery across separate facilities’ [55, 56, 58, 62, 69]. Two articles described how to ‘expand excellent services across geography [55, 69]. Lastly, five articles elaborated on implementing E-health services for patient engagement, referring to the VBHC component ‘building an enabling information technology platform’ [55, 57, 60, 69, 72].

Only 22 of the 40 empirical studies evaluated the implemented VBHC components. Eighteen studies measured the effects primarily quantitatively [9, 14, 15, 18, 19, 24–26, 32–35, 39–42, 44, 50] two qualitatively [8, 29] and two studies combined quantitative research methods with a qualitative approach [17, 36], using a mixed-methods design. The studies that measured the effects of implementing ‘measure outcomes and costs for every patient’ (*N* = 4) reported a decrease in healthcare costs [32], as well as an increased number of patients that felt that the provider spent enough time with them [15]. The studies that measured the effects of implementing both ‘measure outcomes and costs for every patient’ and ‘organize into IPUs’ (*N* = 6) reported: increased patient satisfaction [40], decreased length of stay [40, 44], increased quality of life [18], reduced costs [14, 26] and decreased healthcare utilization [35]. The studies (*N* = 2) that measured the effects of ‘organize into IPUs’, showed an increase in quality adjusted life years and financial benefit for the provider [19]; and a decrease in pre-operative MRIs [33]. The implementation of bundled payments (*N* = 2) led to a decrease in patients admitted to skilled nursing facilities [50], total medical expenditure [34] and length of stay [50]. Two articles measured the effect of ‘measure costs and outcomes for every patient’ in combination with ‘integrate care delivery across separate facilities’ and found an increase in patient satisfaction [25], and an increased number of primary care visits [42]. One study evaluated the implementation of ‘measure outcomes and costs for every patient’ and ‘building an enabling information technology platform’ and reported increased positive experiences with the implementation of PROMs [36].

The remaining articles evaluated the VBHC component that was implemented qualitatively (*N* = 2), applying methods such as semi-structured interviews or focus groups. One of the qualitative studies implemented ‘building an enabling information technology system’ and found that it improved coordination and optimized levels of care [29]. The qualitative analyses in the two mixed-methods studies found that dedicated resources, change of culture and improved knowledge and awareness about VBHC are crucial for implementation [36]. Furthermore, patients experienced better doctor-patient communication after VBHC implementation [17].

### VBHC implementation strategies and their effects

Implementation strategies were described in 19 of the empirical articles. Seven studies focused on educating employees a nd patients [41], including training sessions [7, 17, 37, 41, 50] or symposiums [36] explaining the goals of the VBHC component and teaching how to work according to newly introduced VBHC principles. Another frequently (*N* = 11)[18, 20, 21, 23, 24, 32, 36–38, 46, 47] described implementation strategy was creating interprofessional or multidisciplinary teams, consisting of employees with different professional backgrounds. These project teams, also referred to as taskforces [32], met regularly and were responsible for the implementation of VBHC components such as ‘organize care into IPUs’ or ‘measure outcomes and costs for every patient’. Other strategies described were: making use of a pilot in the first phase of implementation (*N* = 5)(17, 20, 21, 36, 37), including external consultants (*N* = 2)[20, 21], or creating a new position: the chief medical officer, whose task was to drive change towards improved quality and lowered costs [47].

The non-empirical articles proposed a variety of strategies to implement VBHC, see Supplementary Table S3. Similar to the empirical articles, patient and healthcare professional education was an often proposed implementation strategy (*N* = 6)[53, 56, 60, 63, 66, 68]. Furthermore, creating multidisciplinary task forces that were responsible for VBHC implementation, was proposed multiple times (*N* = 5)[58, 66, 67, 70, 71]. Another strategy was increasing awareness of the VBHC implementation by using of ‘champions’ (*N* = 2), i.e. employees who actively work on promoting VBHC [63, 72]. Creating and enhancing leadership was also considered essential in transforming to VBHC (*N* = 1)[63]. These leaders should demonstrate strong commitment to the implementation and should be visible to all frontline healthcare providers.

Only five empirical studies evaluated the implementation strategies used to implement VBHC components. Two qualitative studies evaluated the implementation strategies used for implementing ‘measure costs and outcomes for every patient’ and ‘organizing care into IPUs’, and found that including patient representatives was key in increasing engagement from physicians [20, 21]. Two other evaluated the implementation strategies for ‘measure outcomes and costs for every patient’ [37, 49]. One evaluated ‘the integration of care across separate facilities’ and reported that providing education to employees and patients was critical in the implementation of VBHC, a lack of awareness and lack of knowledge slows down implementation [7]. Other implementation strategies that were proposed to enhance the implementation of this VBHC component in the same studies were creating active and dedicated leadership, and establishing efficient resource allocation [21].

## Discussion and conclusion

### Key findings and contribution

Porter & Teisberg introduced the idea of VBHC with the aim of increasing patient value which they defined as the ratio of outcomes to costs. This article reviewed the academic literature on a) the conceptualization of VBHC in empirical and non-empirical studies regarding implementation of VBHC, b) the implementation of components of VBHC and their effects, and c) the strategies to implement VBHC and their effects. The present study produces three main findings, which are discussed below.

First, our review identified differences in VBHC conceptualization and a high level of interpretative variability. Some authors conceptualized value in healthcare, without conceptualizing VBHC as an overall concept. Others only defined the goals of VBHC, i.e. increased patient value and decreased healthcare costs. Earlier studies also found that VBHC is often interpreted differently across hospitals, and is highly dependent on local choices [73–75].

Differences in policy and payment between and within countries might contribute to this varying conceptualizations as well as implementation strategies needed regarding VBHC. In publicly funded healthcare systems, for instance, there is a stronger emphasis on strategies taking equitable allocation of limited resources into account. In order to accumulate knowledge, concept clarity is needed to distinguish the concept from other seemingly similar concepts as well as to properly test its construct validity [76].

Second, this study found that hospitals apparently do not approach VBHC as an integrative management strategy. According to most studies, hospitals implement one or two components of VBHC only: ‘measure outcomes and costs for every patient’ and ‘organize care into IPUs’ being implemented most frequently. These findings, together with the steep increase in the number of studies in the recent years, suggest that VBHC runs the risk to become a management fad from which hospitals pick a component that best suits their current management strategies. This leads to a fragmentation of VBHC and complicates studying the effectiveness of VBHC as a strategy. Of course, this is also associated with the conceptual ambiguity in the original work of Porter & Teisberg [6] to start with.

Third, this study revealed that implementation strategies were only rarely described, and evaluated even less. Education is the most frequently mentioned strategy for implementing VBHC, both in the empirical and non-empirical literature, which is in line with earlier research on implementation strategies in healthcare [77]. The limited attention to implementation strategies is unfortunate, as studies in change management unequivocally show that the process of change is an important aspect of organizational change explaining its success or failure [78]. This leads us to conclude with a call for an interdisciplinary approach that integrates insights from healthcare and the wider management research community, which extends the argumentation in earlier studies stating that a broader scientific approach to VBHC is urgently needed [79].

### Limitations

Limitations of the current study are related to the eligibility criteria. First, we included articles that explicitly used the term VBHC or Value-Based Care in the full text with an explicit reference to Porter & Teisberg, as we aimed for taking stock of the studies that were based on their original work. Relevant studies on related concepts such as value-based payment [80], capitated model [81] or accountable care organization [82, 83] were therefore not included. Furthermore, in the title-abstract screening phase we found that a large number of articles used the term VBHC in their keywords, title or abstract, while the main focus of the study was not VBHC. This also underlines the earlier observation that VBHC may have turned into an umbrella construct with a high level of interpretative variability [84].

Second, we included English-language articles only, which may have caused a country bias. For example, we encountered a few studies on VBHC in Läkartidningen, a Swedish medical journal [85–87], that we did not include, because they were written in Swedish.

## Conclusions

This study showed that VBHC has a high level of interpretative variability and is translated differently in local hospital settings. While most hospitals stick close to the ideas of Porter & Teisberg and implement outcome measurements, healthcare costs measurements or IPUs, VBHC is not embraced as an integrative strategy. A common conceptualization of VBHC is urgently needed, in order to have a shared understanding of the application of VBHC and to distinguish it from other broader concepts. Furthermore, this review revealed that only few studies evaluate implementation strategies. These findings generally point at a lack of attention for the managerial aspects of VBHC implementation. Interdisciplinary collaboration in future research on the effectiveness of VBHC implementation is therefore paramount.

## Supplementary Information

**Additional file 1: Supplementary Table S1.** Operationalization of data extraction fields.

**Additional file 2: Supplementary Table S2.** Overview of included empirical studies.

**Additional file 3: Supplementary Table S3.** Overview of included non-empirical studies.

**Additional file 4.** PRISMA Scoping Review Checklist.

## Data Availability

The dataset supporting the conclusions of this article is included within the article.

## Abbreviations

VBHC: Value-Based Healthcare
IPU: Integrated Practice Unit
TDABC: Time-Driven Activity Based Costing
PRISMA-ScR: Preferred Reporting Items for Systematic Reviews and Meta-Analyses extension for Scoping Reviews
MeSH: Medical Subject Heading
PROM: Patient Reported Outcome Measure
USA: United States of America
UK: United Kingdom
NL: Netherlands
