# Supplementary material for: The implementation of value-based healthcare: a scoping review
Source: BMC Health Serv Res. 2022 Mar 1;22:270. doi: 10.1186/s12913-022-07489-2 (PMC8886826; doi:10.1186/s12913-022-07489-2)
Supplement: Supplementary file 1 — Additional file 1: Supplementary Table S1. Operationalization of data extraction fields. [file 12913_2022_7489_MOESM1_ESM.docx]

| Supplementary table S1: Operationalization of data extraction fields | | |
| --- | --- | --- |
| Extraction field | **Operationalization** | **Labels** |
| *Author* | First author |  |
| *Year* | Year of publication |  |
| *Country* | Country where the study took place |  |
| *VBHC conceptualization (1)* | Conceptualization of VBHC as described in the study. |  |
| *VBHC conceptualization (2)* | What part of VBHC is conceptualized, divided into three categories:   - The theoretical approach* towards VBHC, including a conceptualization of value and/or goals in VBHC - Solely the value in VBHC - Solely the goals of VBHC | - Concept - Value - Goals |
| *VBHC implementation* | What is implemented or proposed to be implemented as VBHC |  |
| *VBHC component(s)* | The component(s) from Porter & Teisberg that is/are implemented or proposed to be implemented | - Organize into integrated practice units (IPUs) - Measure costs and outcomes for every patient - Move to bundled payments for care cycles - Integrate care delivery across separate facilities - Expand excellent services across geography - Build an enabling information technology platform |
| *Used implementation strategies* | Implementation strategies that were used to implement VBHC |  |
| *Evaluation focus* | The part that is evaluated, divided into two categories:   - Evaluation of the VBHC implementation - Evaluation of the implementation strategy | - Implementation - Implementation strategy |
| *Study design* | The study design, divided into three categories (84):   - Studies with a quantitative approach - Studies with a qualitative approach - Studies combining a quantitative and qualitative approach | - Quantitative - Qualitative - Mixed-methods |
| *Reported effects* | Effects of the VBHC implementation or the used implementation strategy. Either written in text or reported as an increase (↑) or decrease (↓) |  |
