# Supplementary material for: The implementation of value-based healthcare: a scoping review
Source: BMC Health Serv Res. 2022 Mar 1;22:270. doi: 10.1186/s12913-022-07489-2 (PMC8886826; doi:10.1186/s12913-022-07489-2)
Supplement: Supplementary file 2 — Additional file 2: Supplementary Table S2. Overview of included empirical studies. [file 12913_2022_7489_MOESM2_ESM.docx]

| Supplementary table S2: Overview of included empirical studies | | | | | | | | | |
| --- | --- | --- | --- | --- | --- | --- | --- | --- | --- |
| Author | **Year** | **Country** | **VBHC conceptualization (1)** | **VBHC conceptualization (2)** | **VBHC implementation** | **VBHC component(s)** | **Used implementation strategies** | **Evaluation focus** | **Reported effects**  ***(study design)*** |
| Abdulla et al. (1) | 2012 | USA | *A value-based system with value defined as health outcomes achieved per dollar spent (Abdulla et al., 2012, p.2).* | - Concept | Care pathways | Organize care into integrated practice units (IPUs)  Measuring costs and outcomes for every patient | Not specified | Implementation | VBHC can improve patient outcomes while reducing costs and growing volume within a subspecialty surgical service.  *(quantitative)* |
| Ahluwalia et al. (2) | 2019 | UK | Not specified | Not applicable | Protocolized surgical algorithm | Not applicable | Educating healthcare professionals (via education days, audit meetings, regular case-based discussions and trainings) | Implementation | Number of surgical debridement ↓  LOS ↓  *(quantitative)* |
| Ahluwalia et al. (3) | 2020 | UK | Not specified | Not applicable | Day surgery unit (DSU) pathway  Time driven activity based costing | Organize care into integrated practice units (IPUs)  Measuring costs and outcomes for every patient | Not specified | Implementation | Waiting time ↓  LOS ↓  Financial benefit ↑  Patient satisfaction ↑  *(quantitative)* |
| Bernstein et al. (4) | 2019 | USA | *A strategy to deliver healthcare of both high quality and value, commonly defined as health outcomes achieved per dollar spent (Bernstein et al., 2019, p.121).* | - Concept | PROMIS as part of routine care | Measuring costs and outcomes for every patient | Not specified | Implementation | Feeling that provider spent enough time with them ↑  Likelihood to recommend provider to other patients ↑  Rating of provider by patient ↑  *(quantitative)* |
| Bonde et al. (5) | 2018 | Denmark | *A remedial and paradigmatic strategy where the management and governance of healthcare focuses on patients’ health outcomes in proportion to costs of care, and where healthcare is re‐organized from silos of distinct competencies to a focus on the overall delivery of healthcare services across units and organizations*  *(Bonde et al., 2018, p. 1114).* | - Concept | Set departments free to create indicators for measurement | Measuring costs and outcomes for every patient | Not specified | Implementation | The locally developed indicators facilitated ‘dialogical accountability’, which could be an attractive approach for implementing VBHC  *(quantitative)* |
| Brown et al. (6) | 2019 | USA | Not specified | Not applicable | Morehouse Choice Accountable Care Organization | Integrate care delivery across separate facilities  Measuring costs and outcomes for every patient | Integrate and centralize care coordination  Web accessible communication platform  Centralized health information technology warehouse with interoperability | Implementation | Number of primary care visits ↑  Rates for quality measures ↓  Shared savings ↑  Rates of multiple complex conditions ↑  *(quantitative)* |
| Burnhope et al. (7) | 2020 | UK | *Value-Based Health Care (VBHC) is an evolving model of healthcare delivery aimed at achieving better patient outcomes at lower costs to the healthcare provider (Burnhope et al., 2020, p. 1).* | - Concept | VBHC database to integrate, transform and validate health care data | Measuring costs and outcomes for every patient  Build an enabling information technology platform | Not specified | Not applicable | Not applicable |
| Busari, Duits (8) | 2015 | CUR | Not specified | Not applicable | Roadmap to reform healthcare delivery (3 steps) | Integrate care delivery across separate facilities | Not specified | Not applicable | Not applicable |
| Douglas et al. (9) | 2016 | USA | *The authors define value-based health care as providing highest quality of care and best patient outcomes relative to cost in a cross-collaborative partnership between stakeholders (Douglas et al., 2016, p. 55).* | - Goals | Advanced Practice Nurses | Measuring costs and outcomes for every patient | Multidisciplinary task force | Implementation | Sitter usage ↓  Healthcare costs ↓  *(quantitative)* |
| Dronkers et al. (10) | 2020 | NL | *This concept, which was first described by Michael Porter, claims that improvement in both quality and cost of care can be achieved by understanding and integrating the patient perspective into care (Dronkers et al., 2020, p.3591).* | - Concept | ePRO-based clinical support system | Measuring costs and outcomes for every patient  Build an enabling information technology platform | Pilot  Training volunteers to assist patients with questionnaires | Implementation | Patients experienced better doctor-patient communication and increased efficiency of the consultation using the Healthcare Monitor. Patients felt better prepared and experienced more focus on critical issues.  Quantitative analysis did not show significant differences  *(mixed-methods)* |
| Featherall et al. (11) | 2019 | USA | Not specified | Not applicable | Care pathway | Organize care into integrated practice units (IPUs)  Measuring costs and outcomes for every patient | Not specified | Implementation | Episode of care costs ↓  LOS ↓  Discharges to home ↑  No significant differences in 90-day complications or patient willingness to recommend  *(quantitative)* |
| Gabriel et al. (12) | 2019 | UK | *The value-based healthcare (VBHC) agenda sets out to reorientate our healthcare delivery system. Value in healthcare is defined as outcomes that matter to patients and carers relative to the costs of delivering those outcomes and is measured over the complete care pathway. The VBHC approach describes a stepwise process to deliver this model of care* *(Gabriel et al., 2019, p. 2).* | - Value | Care pathway | Organize care into integrated practice units (IPUs)  Measuring costs and outcomes for every patient | Not specified | Implementation | No significant differences in clinical outcomes  Value care ↑  Pathway costs ↓  *(quantitative)* |
| Goretti et al. (13) | 2020 | Italy | *VBHC is a strategic management framework that maximizes the ratio between health outcomes and costs. Developed in 2006, it is based on three acting principles: (i) building value for the patients; (ii) basing the organization of medical practice on medical conditions*  *and care cycles; (iii) measuring outcomes and economic costs (Goretti et al., 2020, p. 2520).* | - Concept | Organizational pathway | Organize care into integrated practice units (IPUs)  Measuring costs and outcomes for every patient | Multi-professional team meetings with patients included  Establish patient engagement | Implementation | Productivity ↑  Quality of life ↑  *(quantitative)* |
| Groeneveld et al. (14) | 2019 | NL | *The concept of VBHC was developed by Michael Porter and Elisabeth Olmsted Teisberg (2006), who stated that “achieving high value for patients must become the overarching goal of health care delivery, with value defined as the health outcomes achieved per dollar spent (Groeneveld et al., 2018, p. 2).* | - Value - Goals | Patient Reported Outcome Measures (PROMs) | Measuring costs and outcomes for every patient | Not specified | Implementation | Good participation, response rate and retention  *(quantitative)* |
| Kaplan et al. (15) | 2014 | USA | *Determining “value” in health care, defined as outcomes per unit cost, depends on accurately measuring cost. (Kaplan et al., 2014, p. 43).* | - Value | Time driven activity based costing | Measuring costs and outcomes for every patient | Not specified | Not applicable | Not applicable |
| Kurt et al.  (16) | 2019 | Turkey | *The value in health care is defined as the ratio of health results to USD spent (Kurt et al., 2019, p. 2754).* | - Value | Time driven activity based costing | Measuring costs and outcomes for every patient | Not specified | Not applicable | Not applicable |
| Lee et al. (17) | 2020 | Taiwan | *Not specified* | Not applicable | ICHOM standard set for older persons | Measuring costs and outcomes for every patient | Not specified | Not applicable | Not applicable |
| Lichkus et al. (18) | 2019 | USA | *Not specified* | Not applicable | Bundled payment | Move to bundled payments for care cycles | Create a transitional nurse care manager  Clinician and patient education | Implementation | Admission to skilled nursing facilities ↓  LOS ↓  *(quantitative)* |
| Martin et al.  (19) | 2018 | USA | *Not specified* | Not applicable | Time driven activity based costing | Measuring costs and outcomes for every patient | Multidisciplinary team | Not applicable | Not applicable |
| McAlearney et al. (20) | 2018 | USA | *Value-based care – most often interpreted as a function of reducing cost while simultaneously improving quality (McAlearney et al., 2018, p. 4767)* | - Goals | Accountable Care Organization | Integrate care delivery across separate facilities | Education | Implementation strategy | Accountability, population health management and engaged physicians are enabling mechanisms for the cultural shift to value  *(qualitative)* |
| McCray et al. (21) | 2017 | USA | *The goal of value based care is to improve patient care outcomes while reducing costs (McCray et al., 2017, p. 58).* | - Goals | Breast cancer care pathway | Organize care into integrated practice units (IPUs) | Not specified | Implementation | Pre-operative MRIs ↓  *(quantitative)* |
| Miao et al. (22) | 2019 | China | *The central goal of value-based healthcare system is to maximize health outcomes with as little medical cost as possible (Miao et al., 2019, p. 246).* | - Goals | Outpatient expenditure reimbursement ratio | Move to bundled payments for care cycles | Not specified | Implementation | Total medical expenditure reimbursement ↓  Per capita annual total out-of-pocket expenditure ↓  Diastolic blood pressure ↓  *(quantitative)* |
| Miettinen & Tenhunen. (23) | 2020 | Finland | *Value is defined as the health outcomes achieved per resources used (Miettinnen & Tenhunen, 2020, p. 572).* | - Value | Digital Cancer Follow-Up Application (CFUA) | Build an enabling information technology platform | Not specified | Implementation | The CFUA improves coordination and optimizes care levels  *(qualitative)* |
| Montesinos Gálvez et al.(24) | 2020 | Spain | *VBHC is a new sanitary paradigm, born from technological advancements, scientific innovation, and changes in patient health culture. It involves a break away from traditional medicine, based on the quantity of services provided, to one based on the value of these services (*Gálvez *et al., 2020, p. 2).* | - Concept | Organizational innovation model based on Advanced Practice Nursing with Ostomies (APN-O) | Organize care into integrated practice units (IPUs) | Not specified | Implementation | Cost difference of 136.99 euros  Quality-Adjusted Life Years ↑  *(quantitative)* |
| Nilsson et al. (25) | 2017 | Sweden | *The model for VBHC is based on*  *three principles: creating value for the patients; basing*  *the organization of medical practice on medical conditions*  *and care cycles; and the measurement of medical*  *outcomes and costs (Nilsson et al., 2017, p. 1).* | - Concept | Interdisciplinary teams defining and using outcome measurements | Organize care into integrated practice units (IPUs)  Measuring costs and outcomes for every patient | Pilot project  Multidisciplinary teams  External consultants  Include patient representatives  Active leadership from management | Implementation strategy | Paying attention to the patients’ voice increased engagement from physicians and care providers  *(qualitative)* |
| Nilsson et al. (26) | 2018 | Sweden | *The model for VBHC is based on three principles: firstly, the overall goal of achieving as*  *much value as possible for the patient when delivering healthcare, secondly, basing the organization of healthcare on the patients’ medical conditions and full care cycles and, thirdly, measuring both health outcomes and the costs of delivering such outcomes. Value for the patients, i.e. “what matters to patients”, is defined as “the health outcomes achieved per dollar spent”. (Nilsson et al., 2017, p. 3).* | - Concept | Interdisciplinary teams defining and using outcome measurements | Organize care into integrated practice units (IPUs)  Measuring costs and outcomes for every patient | Pilot project  Multidisciplinary teams  External consultants  Include patient representatives  Active leadership from management | Implementation strategy | Resource allocation supports implementation, anchoring creates engagement and dedicated development-oriented leadership with power of decision.  *(qualitative)* |
| Pelt et al. (27) | 2018 | USA | Not specified | Not applicable | Real-time cost accounting tool | Organize care into integrated practice units (IPUs)  Measuring costs and outcomes for every patient | Appointing a chief medical officer  Multidisciplinary team | Not applicable | Not applicable |
| Ramsdal & Bjorkquist (28) | 2019 | Norway | *The board’s decision stated that this perspective was based on a normative theoretical*  *operational concept formulated by Stabell and Fjeldstad (1998). Accordingly, the hospital would be organized in line with ‘value chain’, ‘value shop’ and ‘value*  *network’ models (Ramsdal & Bjorkquist, 2020, p. 1719).* | - Concept | A value chain, a value shop, a value network (Stabell/Fjeldstad principles) | Not specified | Not specified | Implementation | Organizing a hospital around a business model such as VBHC is not by definition opposing dominant professional values of health care. As a blend of both, the value concept appeared as a hybrid organization  *(qualitative)* |
| Reilly et al. (29) | 2020 | USA | *A framework for restructuring our health-care systems with the goal of providing better outcomes for patients at lower cost (Reilly et al., 2020, p. 1849).* | - Concept | Value dashboard | Measuring costs and outcomes for every patient  Build an enabling information technology platform | Not specified | Not applicable | Not applicable |
| Van Deen et al. (30) | 2017 | USA | *VBHC is considered to be the solution that will improve quality and decrease costs in healthcare (van Deen et al., 2017, p. 331).* | - Goals | Highly coordinated care pathways | Organize care into integrated practice units (IPUs)  Measuring costs and outcomes for every patient | Introducing task differentiation | Implementation | Healthcare utilization ↓  *(quantitative)* |
| Van Egdom et al.(31) | 2019 | NL | *Value-based healthcare aims to improve the quality of care delivered by measuring and improving outcomes that reflect value instead of volume (van Egdom et al., 2019, p. 1163).* | - Goals | Breast Cancer PROMs and provider  reported outcomes | Organize care into integrated practice units (IPUs)  Measuring costs and outcomes for every patient  Build an enabling information technology platform | Pilot  Institutional dedication  Multidisciplinary team  Education (symposia) | Implementation | Positive experiences with the implementation of PROMs ↑  Dedicated resources, change of culture and practice, and improved knowledge and awareness about VBHC are essential for implementation  *(mixed-methods)* |
| Van den Hoven et al. (32) | 2020 | NL | Not specified | Not applicable | Patient Reported Outcome Measures (PROMs) | Measuring costs and outcomes for every patient | Not specified | Not applicable | Not applicable |
| Van Veghel et al. (33) | 2016 | NL | *This methodology is in line with the Value-Based Healthcare (VBHC) theory,*  *which describes that the patient-relevant outcomes should be evaluated based on the full cycle of care for the patient’s medical condition, rather than comparing isolated interventions (van Veghel et al., 2016, p. 1661).* | - Concept | Patient Reported Outcome Measures (PROMs) | Measuring costs and outcomes for every patient | Facilitate an environment of learning  Multidisciplinary team  Transparent publication of outcomes | Not applicable | Not applicable |
| Van Veghel et al. (34) | 2018 | NL | *Value-based healthcare (VBHC), aiming at maximizing patient value by focusing on outcomes and costs, is considered a strategy for*  *solving these problems (van Veghel et al., 2018, P. 371).* | - Concept | Outcome-based purchasing contract | Measuring costs and outcomes for every patient | Multidisciplinary team | Implementation | Improvements in quality may be ascribed to VBHC implementation  *(quantitative)* |
| Van Veghel et al. (35) | 2020a | NL | Not specified | Not applicable | Outcome based quality  improvement circle | Measuring costs and outcomes for every patient | Group meetings: kick off, in-between meeting, final meeting | Implementation  strategy | The actual use of outcomes to improve quality of care is limited. Roles and responsibilities are not yet formalized. Lack of multidisciplinary steering.  *(mixed-methods)* |
| Van Veghel et al. (36) | 2020b | NL | *VBHC is a promising strategy to increase patient value (van Veghel et al., 2020, p. 1).* | - Concept | Regional integration of healthcare delivery systems and different interventions to improve clinical outcomes and patient satisfaction | Measuring costs and outcomes for every patient  Integrate care delivery across separate facilities | Daily discussion sessions  Multidisciplinary meetings  Include consultant resources  Improve guideline adherence | Implementation | Patient satisfaction ↑  *(quantitative)* |
| Withers et al. (37) | 2020 | UK | *Value-based healthcare*  *(VBHC), which aims to maximise the value of care provided for patients within available resources. It has been defined as the health outcomes achieved per dollar of cost (Withers et al., 2020, p. 2).* | - Goals - Value | Patient Reported Outcome Measures (PROMs) and Patient Reported Experience Measures (PREMs) | Measuring costs and outcomes for every patient | Multidisciplinary teams  Clinical leads | Not applicable | Not applicable |
| Yu et al. (38) | 2016 | USA | *They define value in healthcare as the outcomes achieved per dollar spent (Yu et al., 2016, p. 1962).* | - Value | Time driven activity based costing | Measuring costs and outcomes for every patient | Not specified | Not applicable | Not applicable |
| Yu et al. (39) | 2017 | USA | *Value in healthcare is defined as the ratio of health outcomes achieved per dollar spent (Yu et al., 2017, p. 1045).* | - Value | Time driven activity based costing | Measuring costs and outcomes for every patient | Not specified | Not applicable | Not applicable |
| Zipfel et al. (40) | 2019 | NL | *The goal of value-based healthcare (VBHC) is to reorganize health care in order to increase value for patients. ‘Value’ in VBHC is defined as patient-relevant health outcomes relative to costs. Porter suggests that this goal can be achieved by measuring outcomes and costs per medical condition, which will allow for the identification of variation in outcomes across the full cycle of care (Zipfel et al., 2019, p. 2).* | - Goals - Value | Outcome measurement implementation with the Implementation of Change Model (ICM) | Measuring costs and outcomes for every patient | Pilot  Analysis through questionnaires  Brief implementation plan to employees  Evaluation meeting  Multidisciplinary team  Training  Continuous feedback | Implementation strategy | Application of the ICM facilitates successful implementation of quality- improvement initiatives within VBHC.  *(qualitative)* |
