# Supplementary material for: The implementation of value-based healthcare: a scoping review
Source: BMC Health Serv Res. 2022 Mar 1;22:270. doi: 10.1186/s12913-022-07489-2 (PMC8886826; doi:10.1186/s12913-022-07489-2)
Supplement: Supplementary file 3 — Additional file 3: Supplementary Table S3. Overview of included non-empirical studies. [file 12913_2022_7489_MOESM3_ESM.docx]

| Supplementary table S3: Overview of included non-empirical studies | | | | | | | |
| --- | --- | --- | --- | --- | --- | --- | --- |
| Author | **Year** | **Country** | **VBHC conceptualization** | **Conceptualized part of VBHC** | **Proposed VBHC implementation** | **VBHC component(s)** | **Proposed implementation strategies** |
| Abicalaffe, Schafer (59) | 2020 | Brazil | Not specified | Not applicable | Outcome measurement system | Measuring costs and outcomes for every patient  Move to bundled payments for care cycles | Outsource the implementation to a third-party company |
| Baggaley (56) | 2020 | UK | *At its core, VBHC is a way of*  *driving quality improvement. Unlike existing quality improvement projects, which tend to be stand-alone initiatives, VBHC aims to improve care across whole services or organisations (Baggaley, 2020, p. 198).* | - Goals | Condition-specific IPUs | Organize care into integrated practice units (IPUs)  Measuring costs and outcomes for every patient  Integrate care delivery across separate facilities | Workshops |
| Bauer (60) | 2018 | USA | Not specified | Not applicable | E-health services for patient  engagement | Measuring costs and outcomes for every patient  Build an enabling information technology platform | Patient education |
| Bruch (61) | 2016 | USA | Not specified | Not applicable | Bundled payments | Move to bundled payments for care cycles | Not specified |
| Crowson, Chan (57) | 2020 | CAN | *[…] value-based care models, which are centered on the principle that optimal health care value is achieved when patient outcomes are maximized per unit cost to deliver those outcomes (Crowson & Chan, 2020, p. 1).* | - Goals | Machine learning | Measuring costs and outcomes for every patient  Build an enabling information technology platform | Not specified |
| Furlough et al. (62) | 2020 | USA | Not specified | Not applicable | Multidisciplinary team focused on the outcomes of importance to patients | Measuring costs and outcomes for every patient  Integrate care delivery across separate facilities | Not specified |
| Gupta et al. (63) | 2017 | USA | Not specified | Not applicable | A high-functioning center of value-based care delivery through honest feedback and internal transparency | Not specified | Make use of ‘champions’  Continuous process improvement training  Value-promoting incentives  Use executive leaders that are visible |
| Ibrahim (64) | 2016 | USA | Not specified | Not applicable | An alternative payment model to incentivize care coordination among healthcare delivery to effectively support longer episodes of care | Move to bundled payments for care cycles | Not specified |
| Jørgensen et al. (53) | 2018 | Denmark | *Therefore, there is a need for introducing ‘value-based healthcare’ defined as the value of patient relevant health outcomes in relation to costs (Jorgensen et al., 2018, p. 1).* | - Value | A pragmatic value-based healthcare model with patient identified goals and patient education | Measuring costs and outcomes for every patient | Patient education |
| Keswani et al. (66) | 2016 | USA | Not specified | Not applicable | Integrated Practice Unit | Organize care into integrated practice units (IPUs) | Education (via training)  Multidisciplinary team |
| Keswani et al. (65) | 2018 | USA | Not specified | Not applicable | Time-driven activity based costing | Measuring costs and outcomes for every patient | Not specified |
| Leyton-Mange et al. (67) | 2018 | USA | Not specified | Not applicable | Surgeon value scorecard | Measuring costs and outcomes for every patient | In-person multidisciplinary meetings  Engaging surgeons in creating the implementation |
| Makdisse et al. (51) | 2020 | Brazil | *Value-based healthcare (VBHC) is an emergent health system*  *transformation framework that is gaining global attention in response to increasing healthcare costs and a widespread outcry for replacing fee-for-service payment models, which are seen as a source of overuse and inefficient healthcare delivery (Makdisse et al., 2020, p. 25).* | - Concept | Value management office | Measuring costs and outcomes for every patient  Move to bundled payments for care cycles | Not specified |
| McClellan (68) | 2016 | USA | Not specified | Not applicable | Value-Based Payment programs | Move to bundled payments for care cycles | Health professional education |
| Porter & Lee (69) | 2013 | USA | Not specified | Not applicable | A six-step value agenda for moving to a high-value health care delivery system | Organize care into integrated practice units (IPUs)  Measuring costs and outcomes for every patient  Move to bundled payments for care cycles  Integrate care delivery across separate facilities  Expand excellent services across geography  Build an enabling information technology platform | Not specified |
| Sippo & Nagy (54) | 2014 | USA | *Value in health care is the health outcome per*  *dollar of cost (Sippo & Nagy, 2014, p. 1189).* | - Value | 3 Quality Improvement projects  (1) Digital breast tomosynthesis (2) Indications for breast MRI (3) Coordination of preoperative  imaging guided localization | Measuring costs and outcomes for every patient | Not specified |
| Speerin et al. (58) | 2020 | AU | *Value-based care is touted to achieve the quadruple*  *aim of better health outcomes, better patient and health professional experiences and subsequently improved use of health service resources (Speerin et al., 2020, p. 2).* | - Goals | Models of Care (MoCs) | Organize care into integrated practice units (IPUs)  Integrate care delivery across separate facilities | Multidisciplinary teams |
| Teisberg & Wallace (55) | 2009 | USA | *Value in health care is the improvement in health outcomes relative to the money spent (Teisberg & Wallace, p. 35).* | - Value | (1) Reorganize into clinically integrated teams over the full cycle of care  (2) Measure and report patient outcomes  (3) Enable reimbursement tied to value rather than to quantity of services | Organize care into integrated practice units (IPUs)  Measuring costs and outcomes for every patient  Move to bundled payments for care cycles  Integrate care delivery across separate facilities  Expand excellent services across geography  Build an enabling information technology platform | Not specified |
| Teisberg et al.(52) | 2020 | USA | Value-based health care is a path to achieving the aspirational goals of the  Institute for Healthcare Improvement’s “triple aim”—improving the patient  experience of care, improving the health of populations, and reducing the per capita cost of health care—as well as  improving clinician experience, a fourth  aim that others have proposed *(Teisberg et al., 2020, p. 683).* | - Concept | Strategic framework for VBHC implementation | Organize care into integrated practice units (IPUs)  Measuring costs and outcomes for every patient | Not specified |
| Vetter et al. (71) | 2017a | USA | Not specified | Not applicable | Pass-GO tool + Transitions across Levels of Care (TLC) Service | Measuring costs and outcomes for every patient | Interdisciplinary team huddles |
| Vetter et al. (70) | 2017b | USA | Not specified | Not applicable | Pass-GO tool (Preoperative  Assessment and Global Optimization) | Measuring costs and outcomes for every patient | Interdisciplinary collaboration |
| Winegar et al. (72) | 2018 | USA | Not specified | Not applicable | Measure/collect outcomes | Measuring costs and outcomes for every patient  Build an enabling information technology platform | Surgeon-engagement model with a surgeon-champion |
